# Monitoring brain functioning with evident based IT-technologies with feedback control

**DOI:** 10.1186/1878-5085-5-S1-A100

**Published:** 2014-02-11

**Authors:** Ulyana B Lushchyk, Viktor V Novytskyy, Igor P Babii, Nadiya G Lushchyk, Olena V Babyuk, Andriy O Kovpak, Ivanna I Legka

**Affiliations:** 1 Research Center Veritas, 31 Obolonska Str., of. 9, Kyiv, 04071, Ukraine; 2 The Clinic of Healthy Vessels, 4 Williams Str., Kyiv, 03191, Ukraine; 3 Center for Innovative Medical Technologies Veritas IT Med, 4 Williams Str., Kyiv, 03191, Ukraine

## Scientific objectives

Researches on brain functions, forming of algorithms of neuronets’ functioning and attempt of their active correction are priority in medicine. With IT medical technologies the brain function research like EEG and transcranial electro-stimulation gains new significance and should be revised. Application of methodology for clinical interpretation in the estimation of the brain bioelectric activity allows creating a universal technology for estimation and individually oriented monitoring and correction of brain functions with the purpose of improvement of neurone conductivity, reactivity of cerebral tissues to stimuli, balancing of excitation and inhibition processes [1].

## Results interpretation

During 17 years the Veritas Research Center has investigated sanogenic and pathological reconstructions in the brain bioelectric activity in patients of different type - from healthy to non-curable. 33% of all vascular patients were less curable (hemodynamically marked stenosises of the major arteries, combined cardiovascular insufficiency of II and III stages) and 48% of all psychoneurological patients (apallic syndrome, multiple sclerosis, lateral amyotrophic sclerosis, autism, ICP, epilepsy). The dynamics of brain bioelectric activity testified to large potential in functional activity of the brain, necessity in formation of new algorithms and IT-technologies for monitoring neurodynamics in the treatment process [2,3].

## Outlook and expert recommendations

Applying the IT-technology allows optimizing work of a medical staff, increase efficiency of costs on treatment due to the individually oriented approach to prediction of treatment results and active medical management on the basis of evidential medicine and monitoring of sanogenic reconstructions [2].
